# On a hybrid lossless compression technique for three‐dimensional medical images

**DOI:** 10.1002/acm2.12960

**Published:** 2021-05-07

**Authors:** Boopathiraja Subramanian, Kalavathi Palanisamy, V. B. Surya Prasath

**Affiliations:** ^1^ Department of Computer Science and Applications The Gandhigram Rural Institute Gandhigram Tamil Nadu India; ^2^ Division of Biomedical Informatics Cincinnati Children's Hospital Medical Center Cincinnati OH 45229 USA; ^3^ Department of Pediatrics University of Cincinnati Cincinnati OH USA; ^4^ Department of Biomedical Informatics College of Medicine University of Cincinnati Cincinnati OH USA; ^5^ Department of Electrical Engineering and Computer Science University of Cincinnati OH 45221 USA

**Keywords:** arithmetic coding, lossless compression, object features, radiological images, volume of interest

## Abstract

In the last two decades, incredible progress in various medical imaging modalities and sensing techniques have been made, leading to the proliferation of three‐dimensional (3D) imagery. Byproduct of such great progress is the production of huge volume of medical images and this big data place a burden on automatic image processing methods for diagnostic assistance processes. Moreover, large amount of medical imaging data needs to be transmitted with no loss of information for the purpose of telemedicine, remote diagnosis etc. In this work, we consider a hybrid lossless compression technique with object‐based features for three‐dimensional (3D) medical images. Our approach utilizes two phases as follows: first we determine the volume of interest (VOI) for a given 3D medical imagery using selective bounding volume (SBV) method, and second the obtained VOI is encoded using a hybrid lossless algorithm using Lembel‐Ziv‐Welch Coding (LZW) followed by arithmetic coding (L to A). Experimental results show that our proposed 3D medical image compression method is comparable with other existing standard lossless encoding methods such as Huffman Coding, Run Length Coding, LZW, and Arithmetic Coding and obtains superior results overall.

## 1 INTRODUCTION

Modern medical imaging domain was induced with several new technologies to possess the high quality of medical data. In this context, the magnetic resonance imaging (MRI), computed tomography (CT), positron emission tomography (PET), and single photon emission CT (SPECT) are proven medical imaging modalities and they produce the three‐dimensional (3D) volume of images that are obtained from generating number of slices in a single examination. Each slice is imaged in the same part of the body with some minor level of distance (in mm).^1^ With the advancements in imaging systems, they produce the high spatial resolution and bit depth images for improved quality of images.^2^ This huge amount of medical data is subject to store and transfer for medical diagnostics such as tele‐radiology in telemedicine. This calls for the compression methods for an efficient storage and transmission. Compression methods are naturally classified in two types which are lossy and lossless. The distortion level determines any method to fall under which categories. Lossy methods are irreversible besides the distortion level is also high. Most of the proven lossy compression methods are vector quantization‐based^3^, ^4^ and transform‐based^5^, ^6^ methods. By using wavelet transform,^7^ there can be achieved either lossy or lossless compression with the embedded coder.^8^ As the name itself indicates that lossless compression methods are reversible and there is no information loss (i.e., null distortion level). Lossless compression methods are primarily classified into predictive‐based methods^9^ and statistical methods such as Huffman Coding,^10^ Run Length Coding,^11^ Arithmetic Coding,^12^ Lembel‐Ziv‐Welch Coding (LZW).^13^ Using this type of statistical coding in a hybrid manner proved its efficiency on the 3D domain because of its decorrelation property.^14^

Even though lossy compressed images are visually acceptable, every small detail in the medical image is more important for accurate interpretation and diagnostics, hence, it calls the lossless compression scheme. Moreover, a 3D image is taken for a particular part of a body that contains more amount of statistical redundancy; the above‐mentioned statistical compression is more preferable. An object‐based coding is used in any type of compression method to increase the compression performance. Object refers to the significant region in an image such as region of interest (ROI) in two‐dimensional (2D) images^15^ and volume of interest (VOI) in the case of 3D images. In this paper, we proposed an object‐based compression method to encode and decode the 3D medical images in a lossless manner. The object detection is the principal part of our method that uses bounding volume method on 3D medical image to extract the VOI. In the second phase, the VOI is encoded with a hybrid algorithm that uses LZW and Arithmetic coding. The decoder can reconstruct the actual image as it is with the additionally sent bounding volume information in the encoder. The compression performance of our proposed method is compared with the other lossless compression methods such as Huffman Coding, Run Length Coding, LZW, and Arithmetic Coding. Our method yields better results than the other mentioned lossless compression methods for the 3D medical images.

## 2 MATERIALS AND METHODS

### 2.A VOI coding

Any kind of images especially the medical images are naturally composed by objects. Since each object in an image has its own descriptions and characteristics, they can be classified and used for diverse needs. In terms of medical image compression, clinically important object is known as ROI in the 2D images and in the case of 3D images it is known to be VOI. It may be tumor, brain active regions, and particular tissues or important portions. In our case, the entire object except the background is taken as an important region. As most of the medical images are surrounded with no intensity background, it is known to be a zero‐intensity pixel. It was no more important during the diagnostics and analysis process. By separating the foreground (VOI) from those background, we can increase the compression performance due to its limited pixels. Moreover, those excluded background can be reconstructed in the decompression process to preserve the actual texture and appearance of the original image. During the decompression process, our processed VOI is fused with the non‐object region (background) with the help of intentionally sent non‐object coordinate details in the compressed file. This object‐based coding can improvise certain compression algorithms and leads get high fidelity on clinically significant portions. Even though object‐based coding is the most reasonable solution for image compression, the cost and complexity of these object‐based coding seems to be a constraint in terms of compression efficiency. Some of the related works of such VOI‐based compression methods are discussed below.

A 3D image compression technique which supports the prioritized VOI for the medical images is proposed in Ref. [16]. It presented the scalability properties by means to the lossless construction of images and the optimized VOI coding at any bit rate. A scalable bit stream is created with use of modified 3D EBCOT. A progressive transmission of different VOI is behaved with higher bit rate, in conjunction with the low bit rate background which are essential to identify the VOI in a contextual manner. The demonstrated results achieved higher quality than the 3D JPEG2000 VOI coding method and outperformed the MAXSHIFT and other scalable methods.

Earlier, the segmentation technique‐based image coding presented in Refs. [17, 18, 19] followed a homogenous procedure that involved the regular stages like segmentation, contour coding, and text coding. They needed a sophisticated workflow for extracting the closed contours in the segmentation process. It added a piece of overhead to the image coding systems because these class of codes need to be carried on the lossy level to achieve quality on images. In Ref. [20], a new segmentation‐based VOI detection is proposed. It used seeded region growing method to extract the object. The discontinuity index map was used in the embedded region growing procedure to produce an adaptive scanning process. They overcome the issues of pixel being distorted by generating the low‐dynamic range error which took only most correlated neighbor pixels. Then, the discontinuity index map data parts were subject to encode instead of contour by the Bi‐level Image Experts Group (JBIG). The tested medical images of chest and breast gave better results by 4%–28% than the other classical methods such as direct coding by JBIG, JPEG, hierarchical interpolation (HINT), and two‐dimensional Burg prediction plus Huffman error coding methods.

Object‐based coding proposed by Ref. [21], combined the both shape‐adaptive DWT and scaling based ROI named as SA‐ROI. The shape‐adaptive wavelet transform was used to transform the image samples within the object which are then scaled by certain number of bit‐shifts with further bit‐plane encoder along with the shape information. It outperformed the conventional MAX‐SHIFT ROI coding, scaling‐based ROI Coding, and shape‐adaptive DWT coding. However, it needed shape information overhead with higher computational cost and failed in reconstructing the background.

Deep learning‐based compression schemes are also proposed by the researchers in the past. This deep learning is conceived in the form of neural network in the field of compression. A lossless compression scheme for text compression is discussed in Ref. [22]. It was a two‐phase algorithm which works based on the prediction of long‐term dependencies. Initially, the conditional probability distribution of the given data stream is estimated using recurrent neural network probability estimator block in the first phase. In the second phase, an arithmetic coder block is used to encode and decode those conditional probabilities in order to predict next symbols of the data stream. This work was applied on text and genomic data after being experimented and analyzed with the synthetic data.

Dong et al, in [23] proposed a superresolution convolutional neural network (SRCNN) network to compress the images. It mainly focused on pixel‐level image details and used a three‐layer convolution framework to obtain the low‐resolution images. The weight is computed by doubling the cubic differences of the pixels and used it to encode and decode accordingly.

Long short‐term memory (LSTM)^24^ is a RNN framework proposed by Google and an image compression method based on this is presented in Ref. [25]. It used the neural network‐based full‐resolution image compression. A method in Ref. [26], used a three‐layer convolution neural network which extracted the edge feature map from the outcome of the encoder. Despite the actual region in image, this method mainly focused on the edges of the object and it maintained a predefined compression rate during the training of the network.

A novel image compression algorithm^27^ incorporated the two CNN networks namely, compact convolutional neural network (ComCNN) and reconstruction convolutional neural network (RecCNN) for the encoding and decoding respectively. Its notable back propagation scheme with the quantization rounding function increased the efficiency of the algorithm.

A semantic analysis‐based CNN image compression was proposed in Ref. [28]. The collaborative algorithm based on the application of deep learning fields such as image classification and image compression is used. Initially, convolutional neural network (CNN) is used to semantically analyze the image and obtain the semantic importance map. With the help of the semantic importance map, a compression bit allocation algorithm is applied with the recurrent neural network (RNN) which resulted in improvised image reconstruction quality.

In Ref. [29] introduced an automated method to detect the calcified plaque in intravascular ultrasound (IVUS) which used Rayleigh mixture model, the Markov random field, the graph searching method, and the prior knowledge about the calcified plaque. This method is very optimal than the maual computation of the calcified plaque. In Ref. [30], an adaptive region growing‐based automated framework for detecting lumen and media–adventitia borders in intravascular ultrasound (IVUS) was used as the unsupervised clustering method to detect the lumen border in an image. After a mathematical morphology‐based smoothing, the lumen and MA are efficiently identified and detected.

For the angiogram video sequences, a region‐based wavelet encoder was proposed in Ref. [31]. It uses the SPIHT‐based wavelet algorithm along with the texture modeling and the ROI detection stage. The basic philosophy of greater allocation of available bit‐budget in the ROI was implemented. They indirectly detect the important region by the feature of angiogram imagery where different motion of heart was exploited in the background areas. It gave reasonable improvement than the conventional baseline SPIHT algorithm. But, the accuracy of detected shape of ROI is questionable and it was a dedicated method for angiogram imagery. The above two methods keep the bit map of the shape information throughout the encoding and decoding process. Hence, all of the segmentation‐based object detection methods were needed to carry high cost of reconstruction details such as information shape and other non‐negligible information regarding the segmentation process.

A region‐based medical image compression using High Efficiency Video Coding (HEVC) standard was also proposed.^32^ High Efficiency Video Coding is a prediction‐based technique where interband and intraband predictions are made with the already discriminated nonoverlapping blocks. The difference between the original and predicted block values were transformed using discrete cosine transform (DCT) and discrete sine transform (DST). Then the Context adaptive binary arithmetic coding (CABAC) was used to encode the transform coefficients. The upgradation of this interband and intraband prediction‐based HEVC approach was done to improve the lossless performance of HEVC that was implemented.^33^, ^34^, ^35^ Another object‐based medical image compression technique that used Hybrid Lempel–Ziv–Welch and clipped histogram equalization is proposed in Ref. [36].

As the mathematical morphology and image processing domain has its infrangible bond, they can be used to detect the VOI on images with low cost. A Medical image compression based on region of interest with application to colon CT image was proposed in Ref. [37]. They used the lossless method in ROI and motion‐compensated lossy compression technique in other regions. It was mainly focused on the CT image of human colon. With certain morphological image processing techniques, the colon in the image was segmented to detect ROI. An intensity thresholding was performed in the first step to separate the tissues and the 3D extension of the Sobel's derivative operation was used to extract the colon wall. Then, the morphological 3D grassfire operation was applied for detection of the colon‐wall. This algorithm detects the object in a slice by slice manner. Then, the proposed motion compensated hybrid coding was applied and it outperforms conventional method.

The object‐based coding can improvise certain compression algorithms and leads to get high fidelity on clinically significant portions. Even though object‐based coding is the most reasonable solution for medical image compression, the cost and complexity of these object‐based coding seems to be a constraint in terms of compression efficiency. It is evident that from the perusal of the past literatures explored, some methods suffered by distinguishing the object from background. Shape‐adaptive methods consider only the object but not non‐object details which may also contain some clinically significant details, In the encoder of the shape‐adaptive methods, there is a need to carry the shape information or segmentation details which are treated as an additional overhead in many of the existing techniques.

**Fig. 1:**
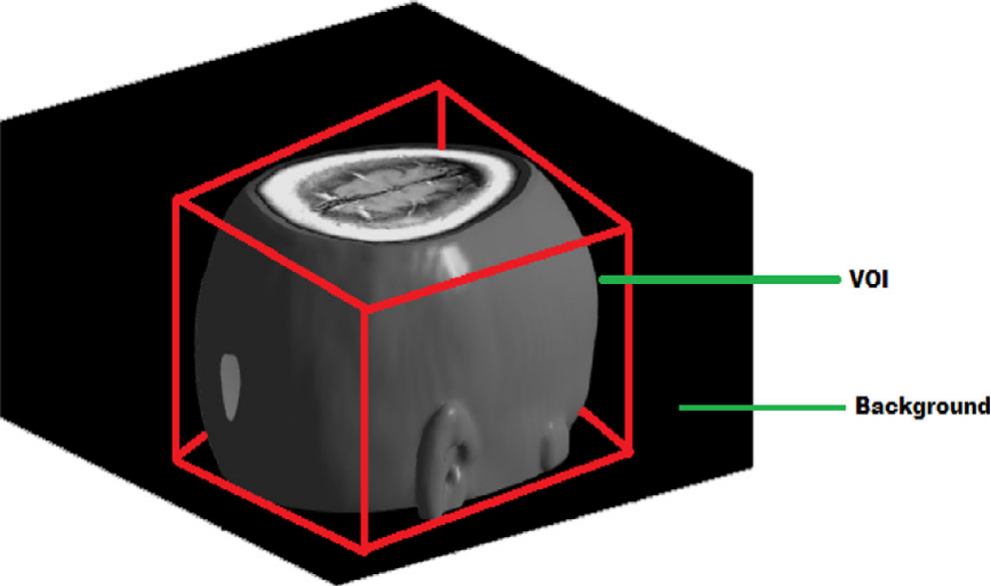
Extracted volume of interest (VOI) using selective bounding volume method (SBV).

To overcome the stated issues, we proposed an automated morphological operation‐based object detection. The VOI is identified and extracted using Selective Bounding Volume (SBV) Method. It is the 3D extension of the Bounding Box method. The algorithm of SBV is given in Algorithm 1. Initially, we apply the Bounding Box operation throughout the volume. Corresponding coordinate value of each slice is recorded and the global value of coordinates are identified as not refusing any active portion in an image slice. Prior to that, our algorithm ignores the blank slices (known as null image slices). In general, these are the foremost and end slices which has no content. Then, the bounding volume is constructed using the confined global coordinates by applying bounding box morphological operation on the volume except the blank slices. The extraction of VOI by SBV method applied on the 3D image is shown in Fig. 1. In Fig. 1, the portion which bounded with the red cube is known as VOI and the regions other than the VOI are called as background. Indeed, this SBV method separates the VOI and background as described in the Algorithm 1 with the denial of the blank slices. Moreover, it is also noted that this method does not discard any active portions in any slices.

Algorithm 1: Selective Bounding Volume (SBV) method.

**Table:** 

***Input***	**:**	*3D Medical Image (X)*
***Output***	**:**	*Extracted VOI*
***Step 1***	**:**	*[x y z] = size (X)*
***Step 2***	**:**	*Declare Variables:* *RowMin, ColumnMin, RowMax, ColumnMax as Integer* *Initialize Variables:* *temp_coordinates [x1, x2, x3, x4] to zero*
***Step 3***	**:**	*For all z* *Search for zero intensity slices* *End* *z1 ← Starting non‐zero Slice* *z2 ← End non‐zero Slice*
***Step 4***	**:**	*For z1 to z2* *Apply Bounding Box operations on each slice* *Update temp_coordinates* *End*
***Step 5***	**:**	*Assign* *RowMin ← Minimum (x1)* *ColumnMin ← Minimum (x2)* *RowMax ← Maximum (x3)* *ColumnMax ← Maximum (x4)*
***Step 6***	**:**	*For k = z1 to z2* *For i = RowMin to RowMax* *For j = ColumnMin to ColumnMax* *VOI ← X (i, j, k)* *End* *End* *End*

### 2.B Lossless compression

Aforementioned, lossless image compression is very opted for medical imaging field. There are standard lossless compression methods that are available in literature.^38^ The basic clue of the lossless compression algorithms is removing the redundancies in any data. As 3D medical images have lot of self‐similar statistical features, these types of compression predominantly work well. The standard lossless compression methods are Huffman Coding, Run Length Coding, LZW, and Arithmetic Coding. This section explains the working principles of above‐mentioned lossless compression methods.

#### 2.B.1 Huffman coding

Huffman coding is one of the standard coding method used for lossless encoding of data.^39^ It is the improvisation algorithm of the basic Shannon's source coding method.^40^ Huffman coding modified the Shannon's source coding by means of developing the unique way of assigning the codewords such that longer codeword is assigned for less frequent symbol and short codeword is assigned for most frequent symbols. First, it determines the probability for each symbol to occur in an input. Then these probabilities are sorted and the lowest symbol probabilities are merged until the algorithm has only two probabilities. The Huffman tree is generated accordingly and the codewords are assigned by traversing the Huffman tree. The data can be easily decoded with help of constructed codewords. The algorithmic steps involved in Huffman Coding are given below.

**Table:** 

***Step 1***	**:**	*Calculate the probability of each and every symbol of the input.*
***Step 2***	**:**	*Then probability of symbols is arranged in decreasing order.*
***Step 3***	**:**	*The lowest probabilities are merged and this process is continued till* *The last two probabilities are left.*
***Step 4***	**:**	*Based on the details obtained from step 3, the Huffman Tree is generated and bits are allocated accordingly.*
***Step 5***	**:**	*By traversing the Huffman tree the original symbols are decoded.*

#### 2.B.2 Run length coding

Run Length Coding is the simplest method for compressing any type of image data. It works based on exploiting the redundancy in an images pixel values.^41^ An ordered pair is recorded in this method such that the pixel intensity value is together with a corresponding consecutive length of this particular pixel. This algorithm is easy to implement and it only worked well for the types of images which have more number of repetitive data such as medical images. The algorithmic steps involved in Run Length Coding are given below.

**Table:** 

***Step 1***	**:**	*Read the pixel continuously from first pixel of an image. If it is the last pixel then exit.*
***Step 2***	**:**	*If the value of the next pixel is same as previous, then count value increment by one. Otherwise, store the pixel value in a new array.*
***Step 3***	**:**	*Finally, the sequence of ordered pair is obtained with the intensity and its corresponding length.*
***Step 4***	**:**	For reconstruction, the empty array with input size is created.
***Step 5***	**:**	*Construct the ith row of compressed image by putting run length value in reconstruct array from compressed array.*
***Step 6***	**:**	*Then, construct* $i+1^{\mathrm{th}}$ *row, then next row, and so on to get the reconstructed image.*

#### 2.B.3 LZW coding

Lembel‐Ziv‐Welch Coding is the dictionary‐based method where number of repetitive image pixels can be identified by the single index.^42^ In the dictionary, it contains the character sequence that was chosen form an image dynamically. An index is assigned to each character sequence on a maximum of 4096 character sequences. It is also known to be a greedy algorithm where the dictionary is modified in each iteration for each new string that is, character sequence. The algorithmic steps involved in LZW Coding are given below.

**Table:** 

***Step 1***	**:**	*The dictionary is initiated such that all input strings have length as one.*
***Step 2***	**:**	*The longest string is identified for the current input symbol with the use of dictionary.*
***Step 3***	**:**	*The dictionary index is emitted this string for output and removed from input*.
***Step 4***	**:**	*Go to next symbol and do the same until all the input symbols are processed*

#### 2.B.4 Arithmetic coding

Arithmetic coding was also working based on the probability of occurrence of symbols nn. It assigns the variable length codeword to the sequence of symbols. A symbol sequence is represented by a real valued interval of [0,1). If the sequence becomes longer then the interval becomes smaller and the number of bits needed to represent those interval is increased. The interval is narrowed while processing each symbol. and finally a particular portion is allocated for certain symbols. The algorithmic steps involved in Arithmetic Coding are given below.

**Table:** 

***Step 1***	**:**	*Range of real numbers are taken as [0,1). This range is divided into subranges according to the symbols in the input. This subrange denotes a real value equal to the source symbol probability.*
***Step 2***	**:**	*Find the subrange for each input symbol that belongs.*
***Step 3***	**:**	*Again, these ranges are subdivided to get the next level subranges.*
***Step 4***	**:**	*Move to the next symbol and do it as same.*
***Step 5***	**:**	*Step 3 and Step 4 is repeated until all the symbols in the input are parsed.*

### 2.C Proposed L to A codec

Since the statistical‐based coding proved its efficiency on better performance for compressing medical images, we provide a hybrid lossless compression method named L to A codec that combinedly uses statistical‐based methods such that LZW algorithm followed by Arithmetic coding for compressing 3D medical images. In this method, initially. the LZW algorithm is applied on certain images and those results are recorded. The results of LZW are sent to the arithmetic encoder. The bit stream obtained after applying the arithmetic encoding is the final compressed file that was sent to the decoder for further decoding processes.

**Fig. 2:**
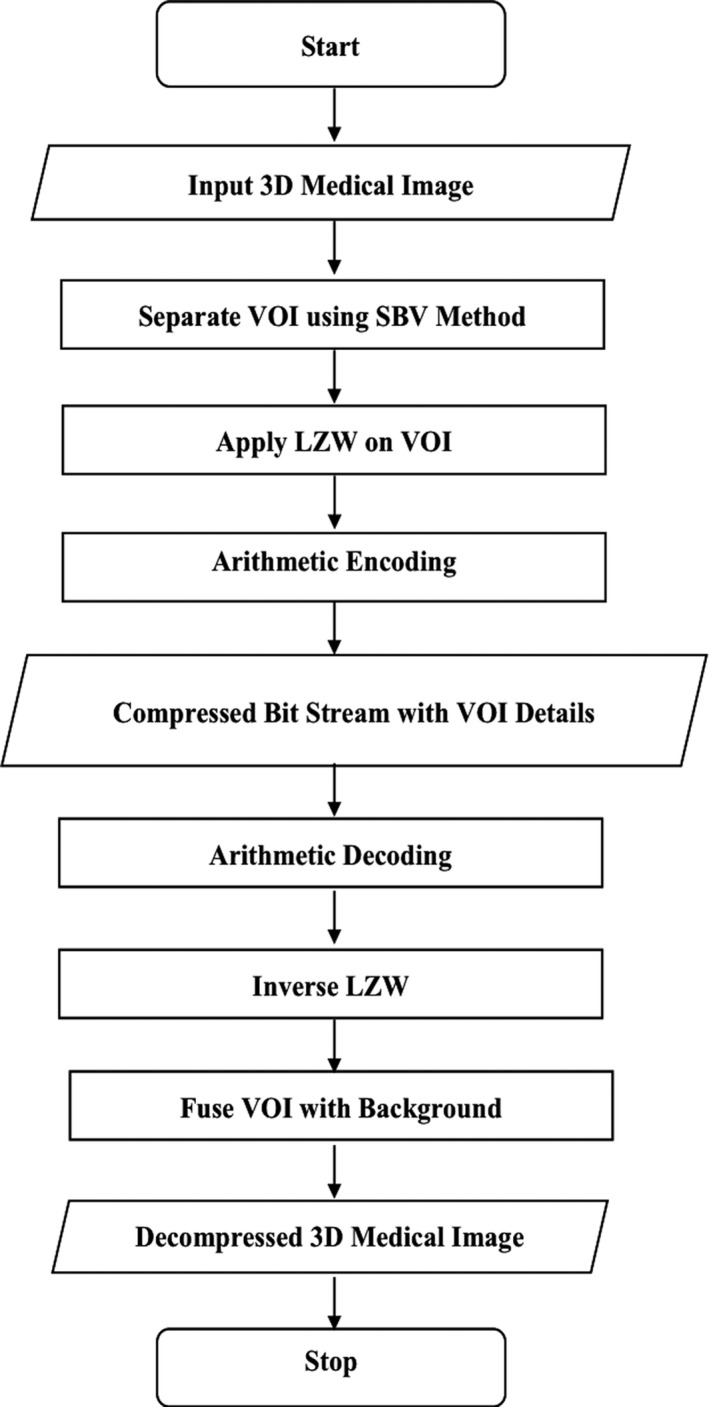
Flow diagram of our proposed three‐dimensional‐volume of interest‐LtoA Method.

The flow chart of our proposed method is depicted in Fig. 2.

The methodology of our proposed method is explained in the following steps.

#### 2.C.1 Encoder

##### Step 1: **input of 3D medical images**

This proposed method is tested with 3D brain volumes which are taken from various online repositories such as the cancer imaging archive (TCIA), internet brain segmentation repository (IBSR), and Radiopedia. Different types of 3D medical images with varying depth dimensions have been tested with this proposed method. All the 3D images tested with the proposed method are 8‐bit depth images.

##### Step2: **extracting the VOI using selective bounding volume (SBV) method**

The VOI extraction in our method separates the foreground from background using SBV algorithm. Initially, we apply the Bounding Box operation throughout the volume. Corresponding coordinate value of each slice is recorded and the global value of coordinates are identified as not refusing any active portion in an image slice. Before that, our algorithm ignores the blank slices that probably are the foremost and end slices which have no content at all known as null image slices. Then the Bounding Volume is constructed using the confined global coordinates by applying bounding box morphological operation on the volume except the blank slices. Furthermore, all the encoder and decoder processes are applied only with VOI. As mentioned earlier, after decoding, the whole image is obtained by fusing the VOI to the background with the Bounding Volume coordinate details sent from the encoder.

##### Step 3: **encoding using L to A encoder**

After extracting the VOI, we apply LZW coding on the extracted VOI followed by an Arithmetic encoding. For that, the results obtained from the LZW coding are sent to the arithmetic encoder which results the compressed bit stream of the given input data (VOI). Besides the compressed bit stream, the coordinate details of the Bounding Volume that are needed to reconstruct the actual 3D volume is also sent on the encoder.

#### 2.C.2 Decoder

##### Step 4: **decoding using L to A decoder**

The decoding process is performed with the obtained compressed bit stream. The inverse operations of the encoder such as Arithmetic decoding followed by the inverse LZW is applied on the compressed bit stream to decompress the VOI. This decompressed VOI data are subject to further processing, which is the reconstruction process.

##### Step 5: **fusing VOI with background**

As the compression and decompression processes are done only in the extracted VOI, they need to fuse the excluded background. Since we already sent the Bounding Volume coordinate details as well as the actual size of the original 3D image volume in the encoder, we first build the empty 3D volume as the actual size of original volume. Then, the decompressed VOI is fused with the built empty volume to get the actual 3D volume. Thus, the compressed 3D medical image volume is decompressed by decoding and fusing processes.

## 3 EXPERIMENTAL ANALYSIS

### 3.A Evaluation metrics

The peak signal to noise ratio (PSNR) and structural similarity index (SSIM) are the qualitative metrics to compare the images. Compression ratio (CR) and bits per pixel (BPP) are other metrics to compare the original image and reconstructed image in terms of compression performance.

#### 3.A.1 Compression ratio (CR)

Compression ratio is defined as number of bits to represent the size of original image to the number of bits to represent the compressed image. Compression ratio can be determined using the following formula.

(1) CR=n1/n2

where, n1— Number of bits in the original imagen2 — Number of bits in the compressed image

#### 3.A.2 Bit rate (BR)/bits per voxel (BPV)

Number of bits are needed to encode the unit of pixel is known as BR. The formula for BR is given below

(2) BR=C/N

where, C — Compressed image bits, N — Total no. of pixels.

In the case of 3D images, the basic unit is known as voxel and hence it is called as bits per voxel (BPV). Bits per voxel denotes the number of bits required per voxel of a 3D where the N denotes the total number of voxels.

#### 3.A.3 Peak signal to noise ratio (PSNR)

The PSNR is the widely used measure to find the quality of the reconstructed image. The following formula is used to calculate the PSNR value.

(3) $$PSNR=10.log10\left(MAX^{2}/MSE\right)$$

where, MAX is the maximum intensity pixel value of the image.

The MSE is the cumulative squared error between the compressed and original image. The following formula is used to find the MSE value.

(4) $$MSE=\frac{1}{\mathrm{MN}}\sum_{y=1}^{M}\sum_{x=1}^{N}{\left[Im\left(x,y\right)‐Im^{\prime }\left(x,y\right)\right]}^{2}$$

#### 3.A.4 Structural similarity index (SSIM)

Structural similarity index is the HVS‐based quality Metric. It is used to measure the similarity between the given original and compressed image. It is the perceptual metric to quantify the image quality degradation. The reference and the processed images are compared through SSIM. Local image structure, luminance, and contrast are analyzed by SSIM metric.

(5) $$SSIM\left(x,y\right)={\left[l\left(x,y\right)\right]}^{\alpha }.{\left[c\left(x,y\right)\right]}^{\beta }.{\left[s\left(x,y\right)\right]}^{\gamma }$$

where,

(6) $$l\left(x,y\right)=\frac{2\mu_{x}\mu_{y}+c_{1}}{\mu_{x}^{2}+\mu_{y}^{2}+c_{1}}$$

(7) $$c\left(x,y\right)=\frac{2\sigma_{x}\sigma_{y}+c_{2}}{\sigma_{x}^{2}+\sigma_{y}^{2}+c_{2}}$$

(8) $$s\left(x,y\right)=\frac{2\sigma_{\mathrm{xy}}+c_{3}}{\sigma_{x}\sigma_{y}+c_{3}}$$

where, $\mu_{x}$ and $\mu_{y}$ are the local means, $\sigma_{x}$ and $\sigma_{y}$ are the standard deviations, and $\sigma_{\mathrm{xy}}$ is cross covariance for images x and y.

## 4 RESULTS AND DISCUSSION

To quantify the performance of this proposed method, randomly selected samples of 3D medical image volumes are tested with this proposed method. The 3D Image 1 is the MRI brain data of 128 × 128 × 27 image, 3D Image 2 is the CT brain data of 256 × 256 × 99, 3D Image 3 is the 256 × 256 × 99 MRI Head Image, the 3D Image 4 to 3D Image 11 have the dimension of 256 × 256 × 63 which were taken from IBSR and the final 3D Image 12 is a PET image dataset of 258 slices (256 × 256 × 298) and all the 3D images are 8‐bit depth images.

Since, this proposed method is constructed as a lossless method, MSE value becomes zero, so that PSNR values tends to infinity (∞) for all the tested 3D image volumes. The compression performance is evaluated through the Compression Ratio (CR) and Bit Rate (BR). Table 1 shows the numeric results of the proposed 3D‐VOI‐LtoA method in terms of Compression Ratio, Bit Rate/BPV, and computation Time. The tested images with their corresponding dimensions are given in a column and the next three columns contain the values of CR, Bit Rate, Encoding, and Decoding time respectively.

**Table 1:** The CR, bit rate, and computation time of the proposed method three‐dimensional‐volume of interest‐LtoA.

3D image	CR	Bit rate/BPV	Computation time (s)	
			Enc.	Dec.
3D image 1	3.22	2.48	19.23	3.17
3D image 2	2.16	3.7	408.01	48.99
3D image 3	1.92	4.17	445.20	50.28
3D image 4	12.57	0.64	38.94	5.58
3D image 5	13.49	0.59	37.94	5.76
3D image 6	15.61	0.51	31.88	5.71
3D image 7	12.15	0.66	40.60	6.16
3D image 8	11.17	0.72	43.50	6.91
3D image 9	14.14	0.57	40.17	6.14
3D image 10	8.21	0.97	64.08	10.23
3D image 11	9.48	0.84	52.95	8.46
3D image 12	13.66	0.59	204.98	29.66

It is evident from the Table 1 that the proposed method need only less number of bits to store the 3D images instead of using 8‐bit depth. Particularly, most of the 3D medical images require only less than two bit rate to store the 3D image. It also can be observed from the Table 1 that the computation time increases slightly when the number of slices in the 3D image increases. Since these are the 3D images, it consumes little more computation time but they are reasonable. Using the SBV for VOI detection and extraction, leads to attain the hike in the CR and obviously reduces the Bit Rate.

**Fig. 3:**
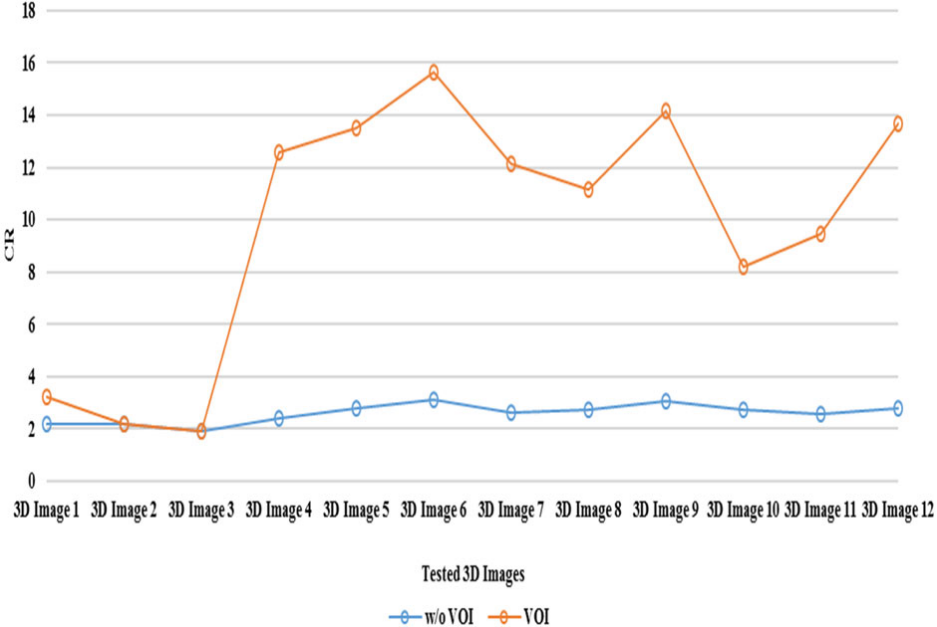
Comparison of volume of interest (VOI) coding vs w/o VOI method in terms of CR.

**Fig. 4:**
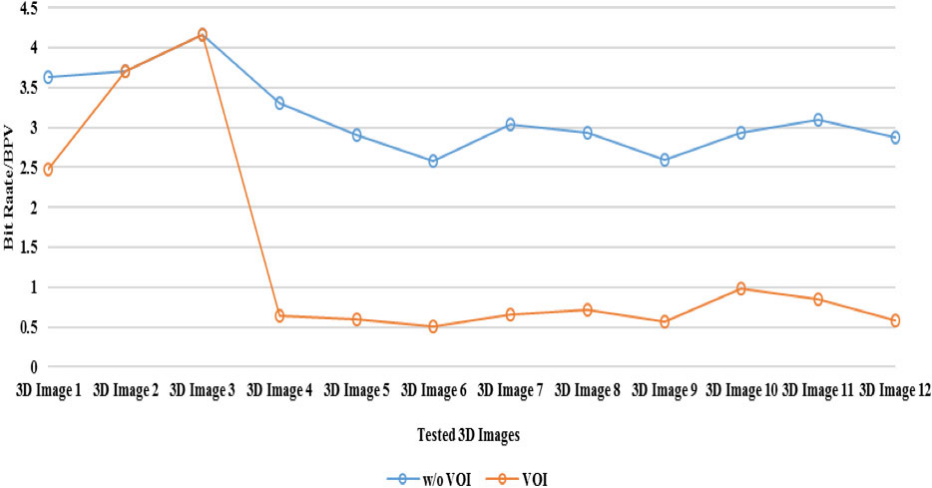
Comparison of volume of interest (VOI) coding vs. w/o VOI method in terms of Bit Rate/BPV.

**Fig. 5:**
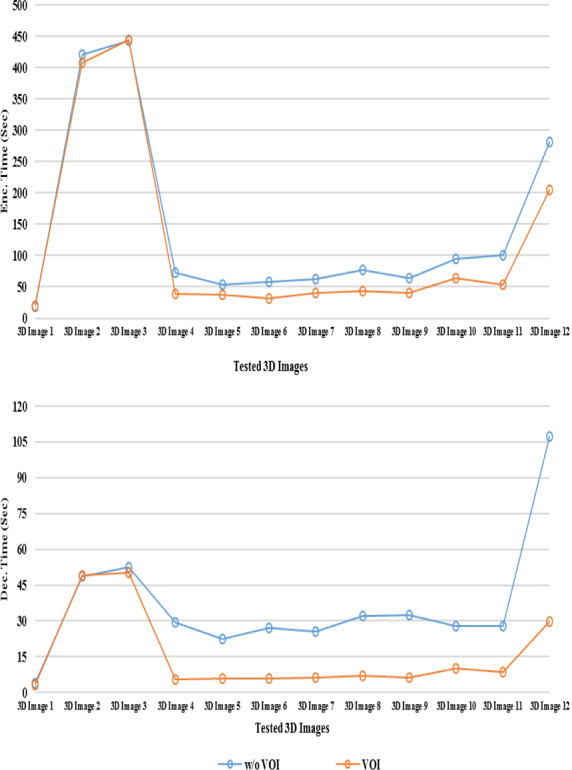
Comparison of volume of interest (VOI) coding versusw/o VOI method in terms of computation time.

Hence this proposed 3D‐VOI‐LtoA method initially detected and extracted the VOI using the SBV method. The LZW compression algorithm is applied only on the extracted VOI. Then the output of the LZW algorithm is sent to the Arithmetic Coding stage to get the compressed bit stream. For the comparative purpose, this proposed method is performed without the VOI detection and extraction phase (w/o VOI). The results of this scenario for the tested 3D medical images will be compared against the actual proposed method. The Figs. 3 to 5 depicted the effectiveness of VOI coding. In these Figures, the results of the proposed method for the various tested 3D volumes between the VOI and without VOI coding phase are presented.

Figure 3 represents the comparison of CR values of the VOI coding and w/o VOI coding; the corresponding Bit Rate and Computation Time between the VOI and w/o VOI are illustrated in Figs. 4 and 5 respectively. This Fig. 3 clearly shows the hike in CR while including the SBV VOI detection and extraction precedence with the encoding phase using L to A Codec. For all the tested 3D medical images, there is an hike of 7.26 on an average for the tested 3D images.

In order to analyze the Bit Rate, Fig. 4 gives the evidence of low bit rate for the VOI coding than the omission of VOI extraction phase. In an average, 1.77 bits are reduced in the VOI coding for the tested images than the w/o VOI coding. As mentioned, the L to A encoding is performed only on the extracted VOI regardless of background. This factor influences the proposed method in order to reduce the computation time (Decoding Time) which is shown in Fig. 5.

**Fig. 6:**
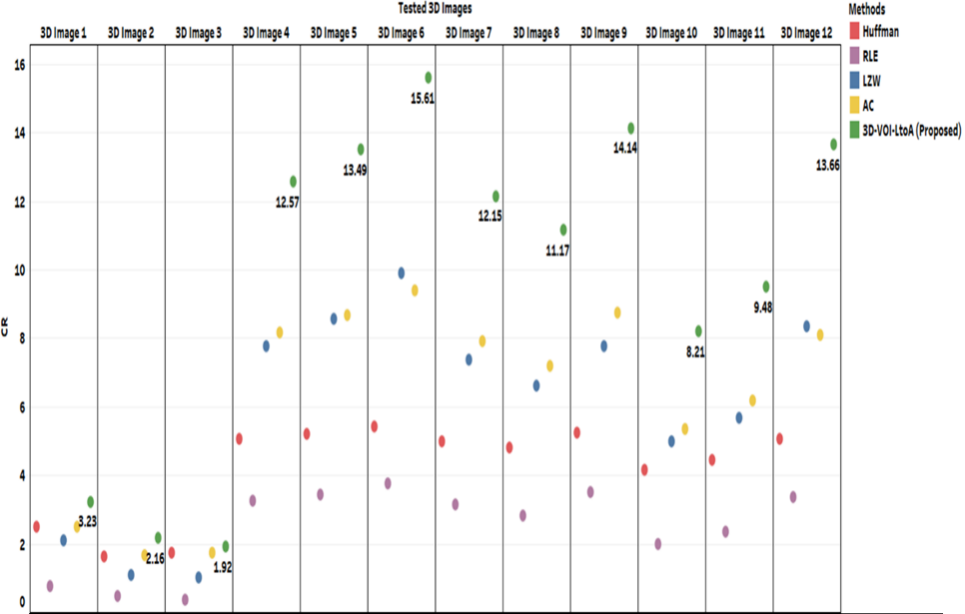
Performance comparison in terms of CR of the Proposed three‐dimensional‐volume of interest‐LtoA Method against the existing methods: Huffman Coding, RLE, Lembel‐Ziv‐Welch coding and AC.

**Fig. 7:**
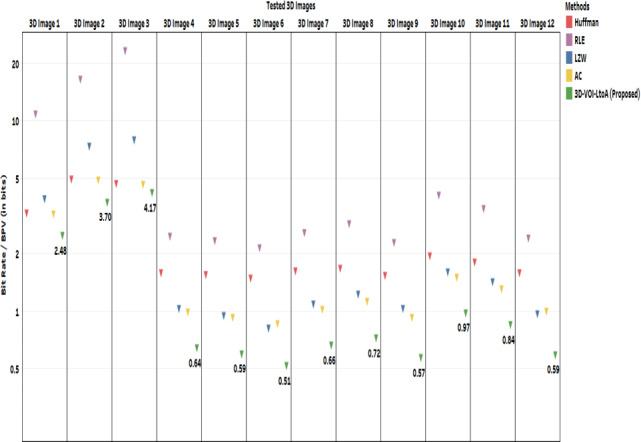
Performance comparison in terms of bit rate/BPV of the Proposed three‐dimensional‐volume of interest‐LtoA Method against the existing methods: Huffman Coding, RLE Lembel‐Ziv‐Welch coding, and AC.

Next we provide comparative results of the proposed method with theexisting. Our proposed method is compared against the existing methods such as Huffman Coding, Run Length Coding, LZW, and Arithmetic Coding. Figs. 6, 7, and Table 2 are representing the comparative results in terms of CR, Bit Rate, and Computation Time respectively. All the methods have no differences in the intensity values of original and decompressed image which means that the lossless compression is achieved. Therefore, all the methods have the null MSE values which lead to PSNR tending to ∞. However, SSIM metric was evaluated and all the tested 3D volumes give the lossless performance as indicating the SSIM value of 1.

**Table 2:** Comparison of computation time of the proposed three‐dimensional‐volume of interest‐LtoA method against the existing methods: Huffman coding, RLE, Lembel‐Ziv‐Welch coding (LZW), and AC.

3D image	Huffman		RLC		LZW		AC		3D‐VOI‐LtoA (proposed)	
	Enc.	Dec.	Enc.	Dec.	Enc.	Dec.	Enc.	Dec.	Enc.	Dec.
1	0.50	13.39	0.02	0.05	8.93	3.20	12.99	14.60	19.23	3.17
2	12.50	293.18	0.47	0.81	126.02	51.40	275.77	322.77	408.00	48.99
3	12.60	314.94	1.00	1.21	171.99	70.85	300.29	295.01	445.20	50.28
4	3.84	72.60	0.09	0.51	36.76	28.08	46.76	56.92	38.94	5.58
5	5.09	62.09	0.12	0.52	28.27	23.04	39.64	43.57	37.94	5.76
6	3.19	72.80	0.06	0.45	34.31	26.95	44.78	49.30	31.88	5.71
7	4.10	66.48	0.07	0.62	29.46	28.01	51.74	56.05	40.60	6.16
8	9.98	84.53	0.30	0.62	36.21	30.88	67.30	71.05	43.50	6.91
9	3.58	77.34	0.08	0.49	38.73	32.90	47.03	48.21	40.17	6.14
10	4.16	104.49	0.37	0.56	40.77	30.22	65.99	81.21	64.08	10.23
11	3.43	83.37	0.31	0.74	44.59	33.01	66.69	76.63	52.95	8.46
12	14.16	289.00	0.44	2.25	148.99	103.84	202.18	230.79	204.98	29.66

From Fig. 6, it is evident that this proposed method produced better results than the other existing methods such as Huffman Coding, Run Length Coding, LZW. and Arithmetic Coding. The size of an object of interest matters when extracting VOI, a shorter VOI leads to high compression performance. In other words, if a longer null intensity background occurs, then we can achieve more compression. For all the 3D volume, our proposed method yields almost double the time of compression ratio than other existing popular methods. Probably, the Bit Rate also very less in our proposed method than the other methods as shown in Fig. 7.

While comparing the computation time, this 3D‐VOI‐LtoA method gives a massive reduction as shown in Table 2. Though the RLE method undergone with the less computation time than the proposed method for the two of the tested images, it need not to taken into account because of the ineffective results of CR and Bit Rate of that method. Thus, our proposed method gives better performance in terms of Compression Ratio, Bit Rate, and Computation Time than all other mentioned existing methods.

**Fig. 8:**
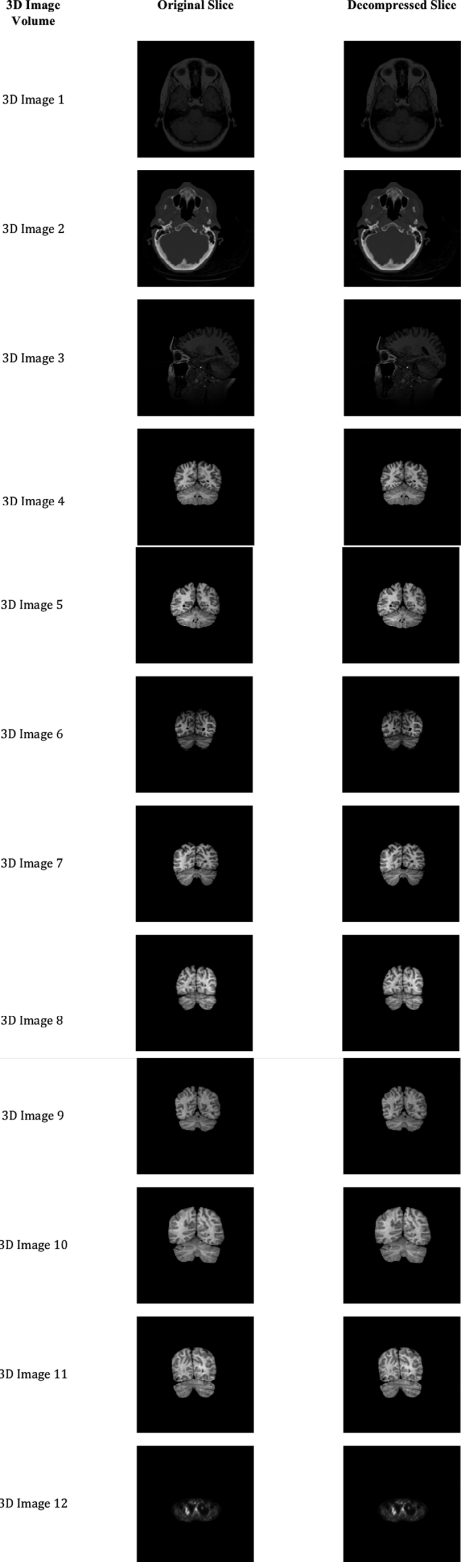
Random slices of the tested three‐dimensional volumes after compression by the proposed method.

Since our proposed method is a lossless compression algorithm, the decompressed images conceive the original data as it is. The resultant images of our proposed method for the tested 3D volumes are depicted in Fig. 8 to express the quality of our algorithm. Column 1 in Fig. 8 indicates the tested volume name and the column 2 and 3 show the random slice of the original and decompressed volume respectively. It is clear from this figure that our method compresses the 3D image volume lossless manner as visually there is no loss of data in the decompressed image.

## 5 CONCLUSIONS

Due to the necessity of compressing the high amount of 3D medical volumes that accompanied with the lossless property for accurate diagnostics process, we have proposed an object‐based hybrid lossless algorithm. The proposed VOI coding shows its effect on increasing the amount of compression ratio as well as reduced bit rate. This method efficiently encodes and decodes the VOI. Using of SBV method to extract, the VOI reduces the complexities of reconstructing the actual 3D image volume with less number of reconstruction details. Moreover, our method yields double the time more compression ratio than the other existing methods on tested 3D Volumes with corresponding low Bit Rate. Importantly, this method reduces the computation time tremendously than the other existing methods.

## CONFLICT OF INTEREST

None.
